# Supplementary material for: Bioinspired structural hydrogels with highly ordered hierarchical orientations by flow-induced alignment of nanofibrils
Source: Nat Commun. 2024 Jan 2;15:118. doi: 10.1038/s41467-023-44481-8 (PMC10761753; doi:10.1038/s41467-023-44481-8)
Supplement: Supplementary file 1 — Supplementary Information [file 41467_2023_44481_MOESM1_ESM.pdf]

Supplementary Information

Bioinspired structural hydrogels with highly ordered hierarchical orientations by flow-induced alignment of nanofibrils

Shuihong Zhu[1,2], Sen Wang[1], Yifan Huang[3], Qiyun Tang[3], Tianqi Fu[1], Riyan Su[4], Chaoyu Fan[1], Shuang Xia[1], Pooi See Lee[2]✉ & Youhui Lin[1,3]✉

[1]Department of Physics, Research Institute for Biomimetics and Soft Matter, Fujian Provincial Key Laboratory for Soft Functional Materials Research, Xiamen University, Xiamen 361005, P. R. China

[2]School of Materials Science and Engineering, Nanyang Technological University 50 Nanyang Avenue, Singapore 639798, Singapore

[3] Key Laboratory of Quantum Materials and Devices of Ministry of Education, School of Physics, Southeast University, Nanjing 211189, P. R. China

[4]National Institute for Data Science in Health and Medicine, Xiamen University, Xiamen 361102, P. R. China

[5]Shandong Huankeyuan Environmental Testing Co., Ltd, Jinan 250013, P. R. China

✉E-mail: pslee@ntu.edu.sg; linyouhui@xmu.edu.cn

Supplementary Figures

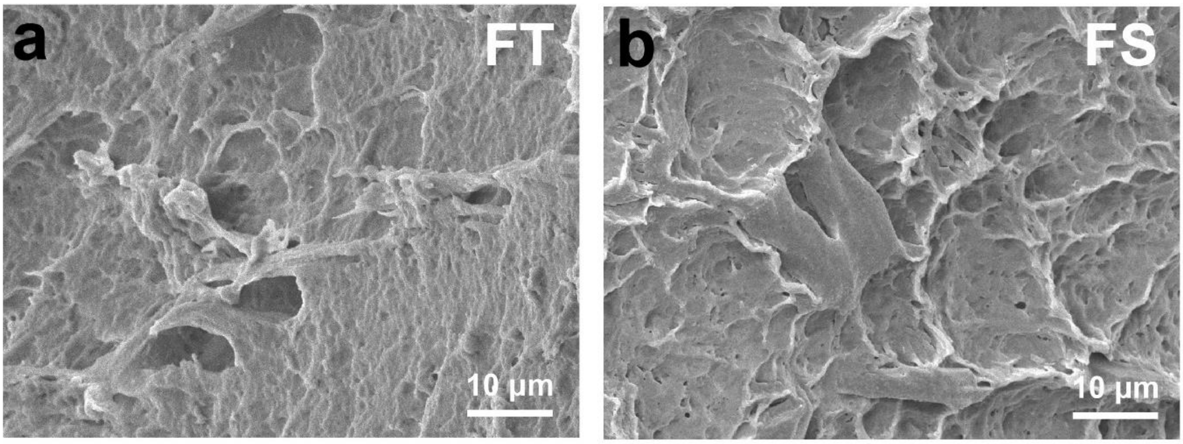

Supplementary Figure 1. SEM images of (a) FT and (b) FS hydrogels with randomly oriented polymer networks.

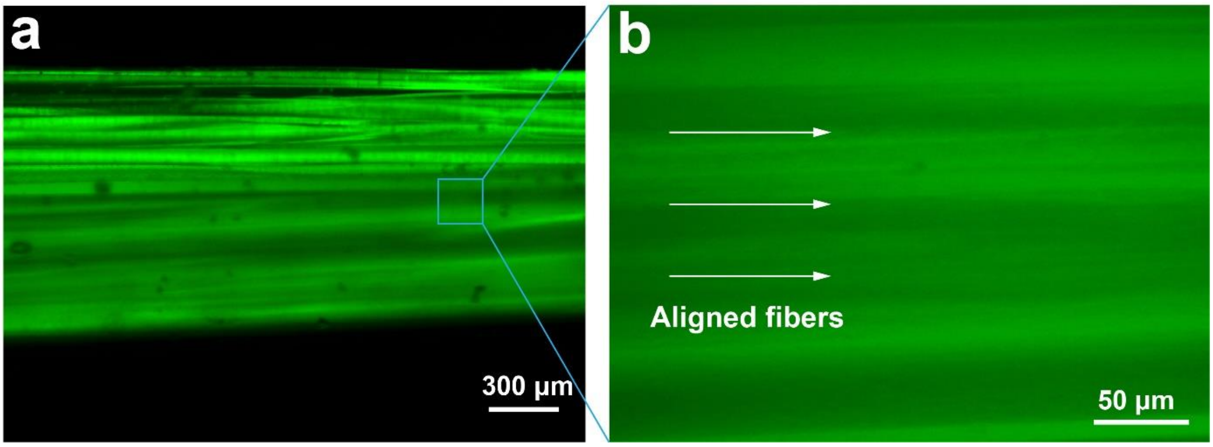

Supplementary Figure 2. Confocal images showing the aligned fibrous microstructures of AFH.

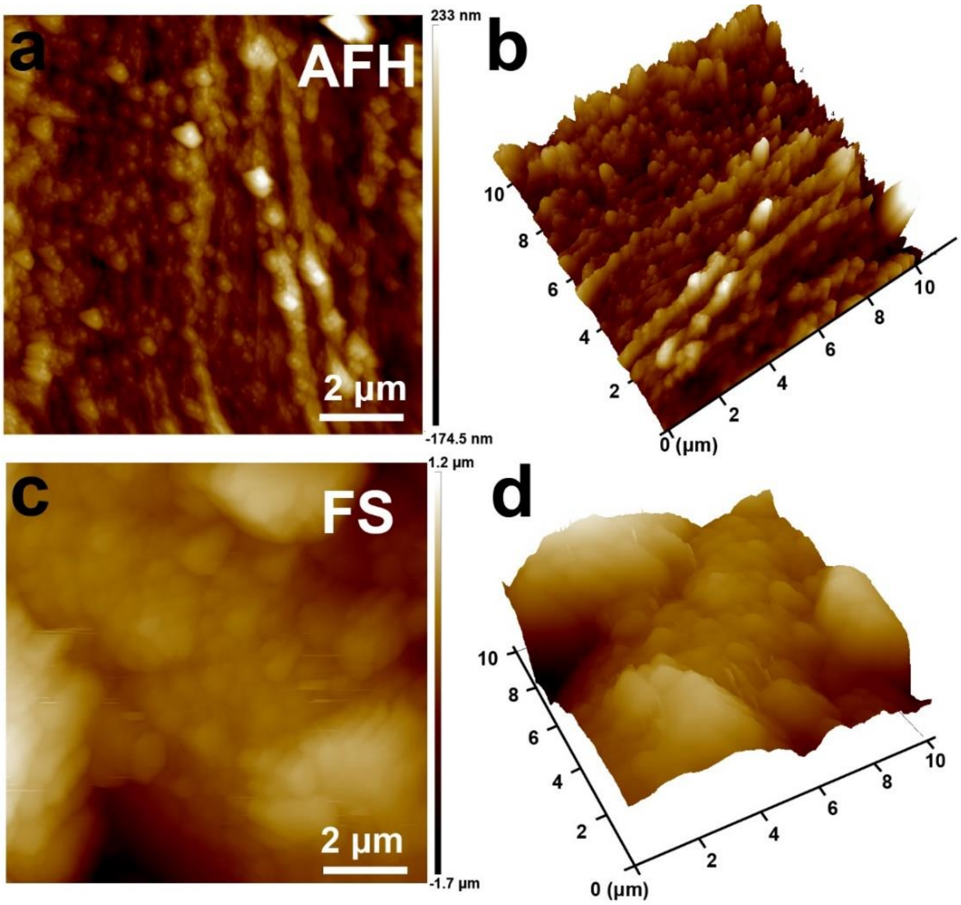

Supplementary Figure 3. (a, b) AFM images showing the aligned fibrous microstructures of AFH. (c, d) AFM images showing homogeneously distributed microstructures of FS.

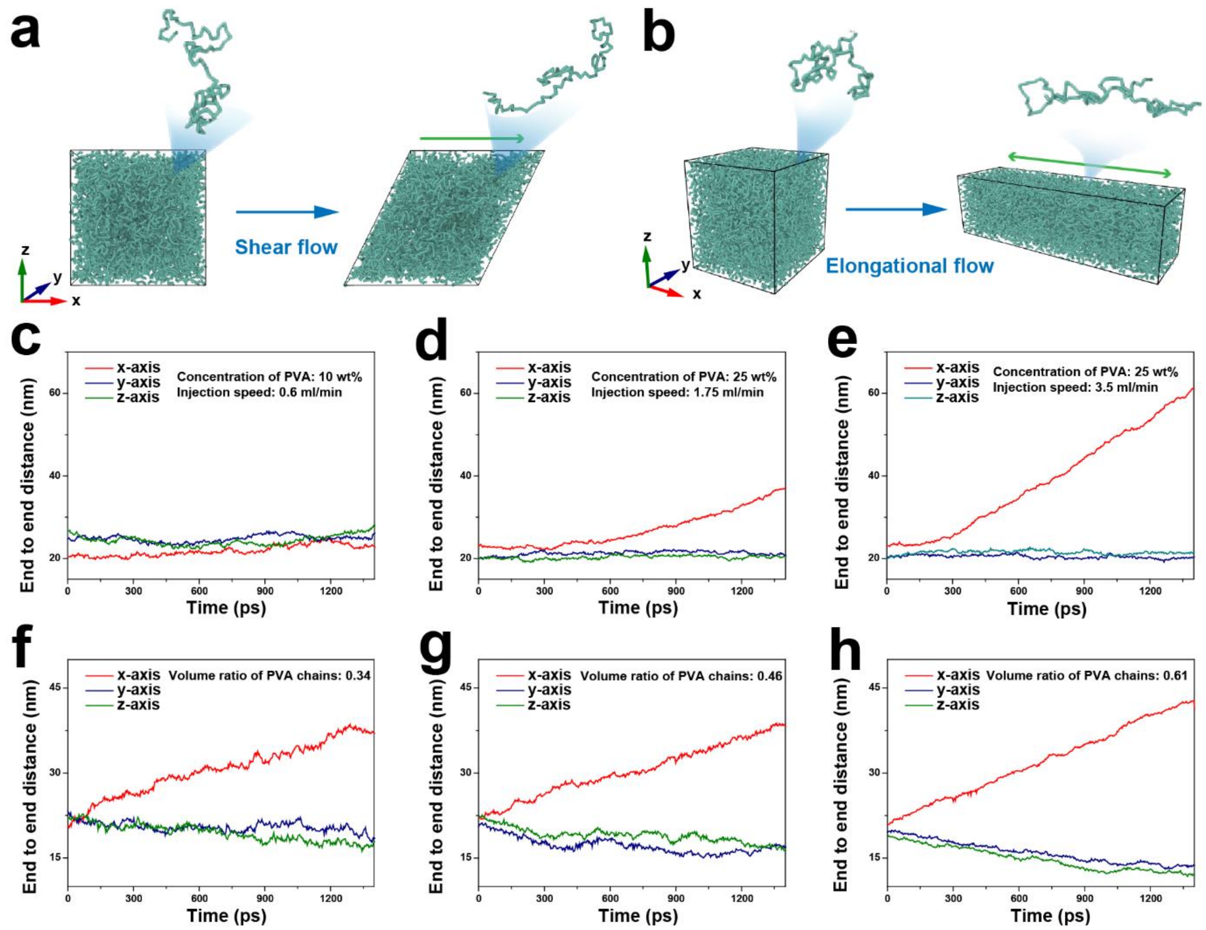

Supplementary Figure 4. Molecular dynamics simulations of PVA chains in (a) the injection process (dominated by shear flow) and (b) the stretching process (dominated by elongational flow). (c-e) The Cartesian coordinate components of the end-to-end distance of PVA chains under shear flow at polymer concentration from 10 wt% to 25 wt%, and injection speed from 0.6ml min^{-1} to 3.5 ml min^{-1}. (f-h) The Cartesian coordinate components of the end-to-end distance of PVA chains under extensional flow at various volume ratios.

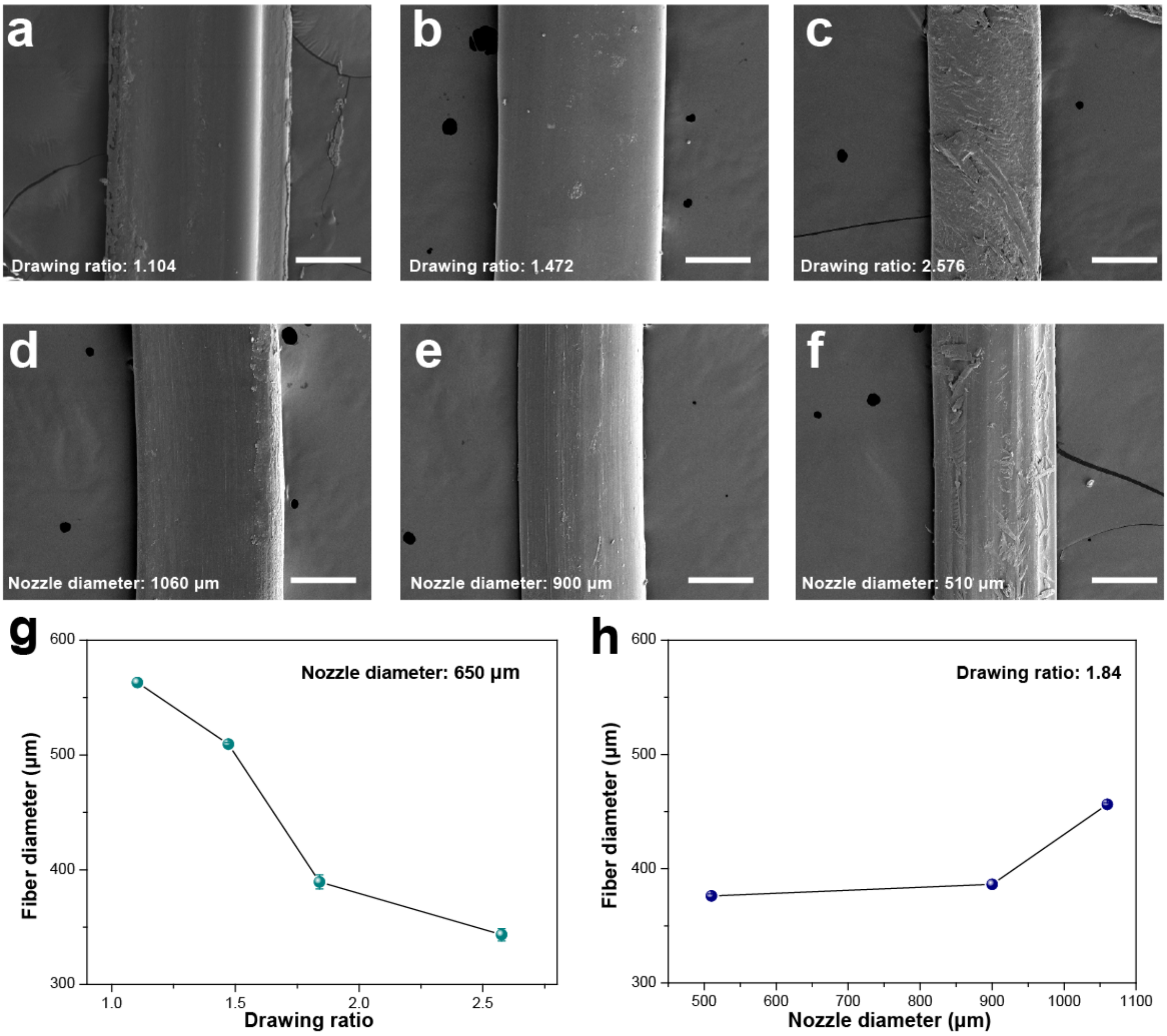

Supplementary Figure 5. Effect of drawing ratio and nozzle diameter on hydrogel fibers during the spinning process. SEM images of hydrogel fibers were obtained with various (a-c) draw ratios and (d-e) nozzle diameters. Scale bar = 200 μm. Average results for fiber diameter at different (g) draw ratios and (h) nozzle diameters. Fiber diameter data are presented as mean values ± SD, n = 3 independent samples.

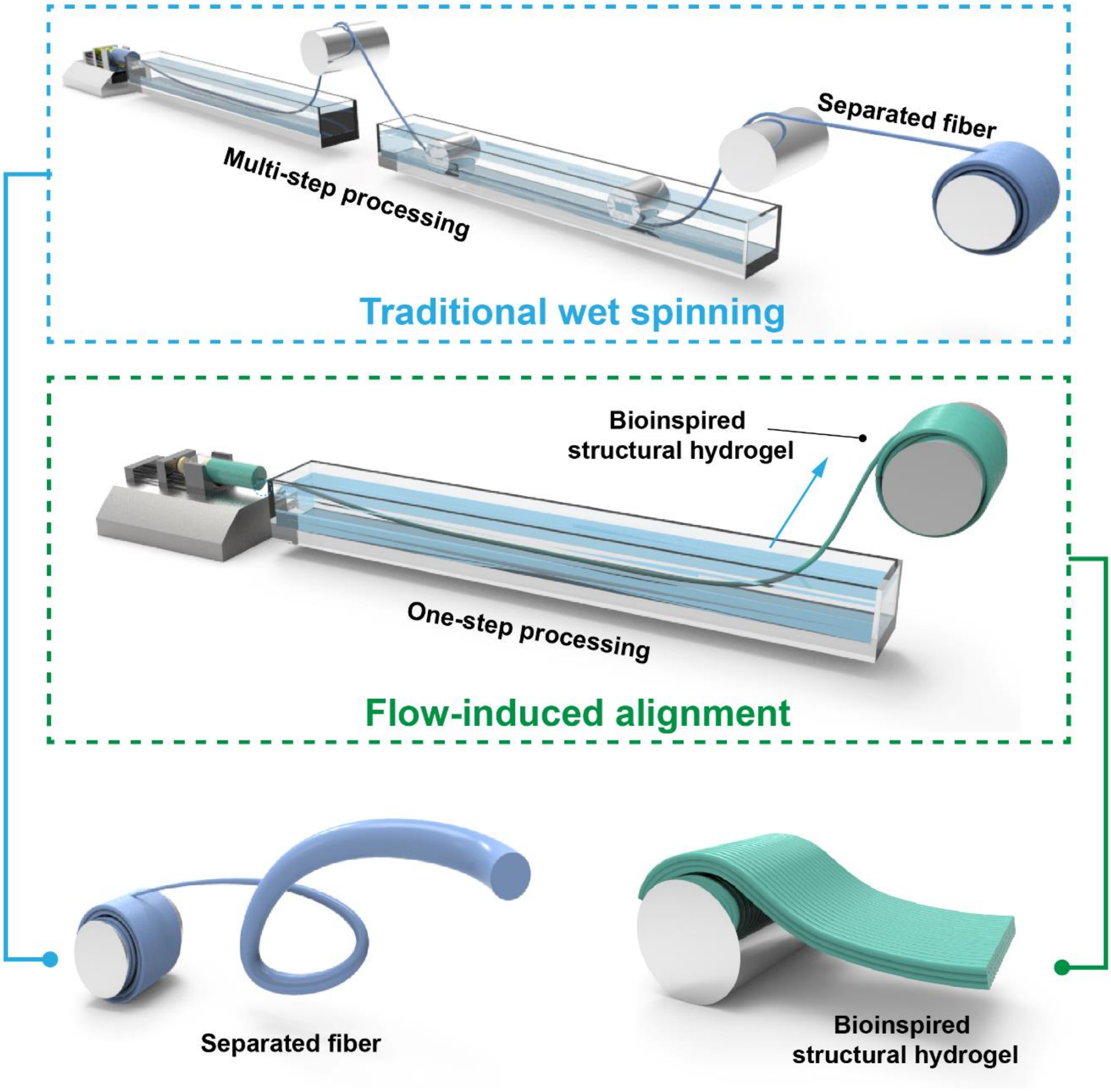

Supplementary Figure 6. Comparison between the traditional wet spinning method and flow-induced alignment strategy proposed in this work.

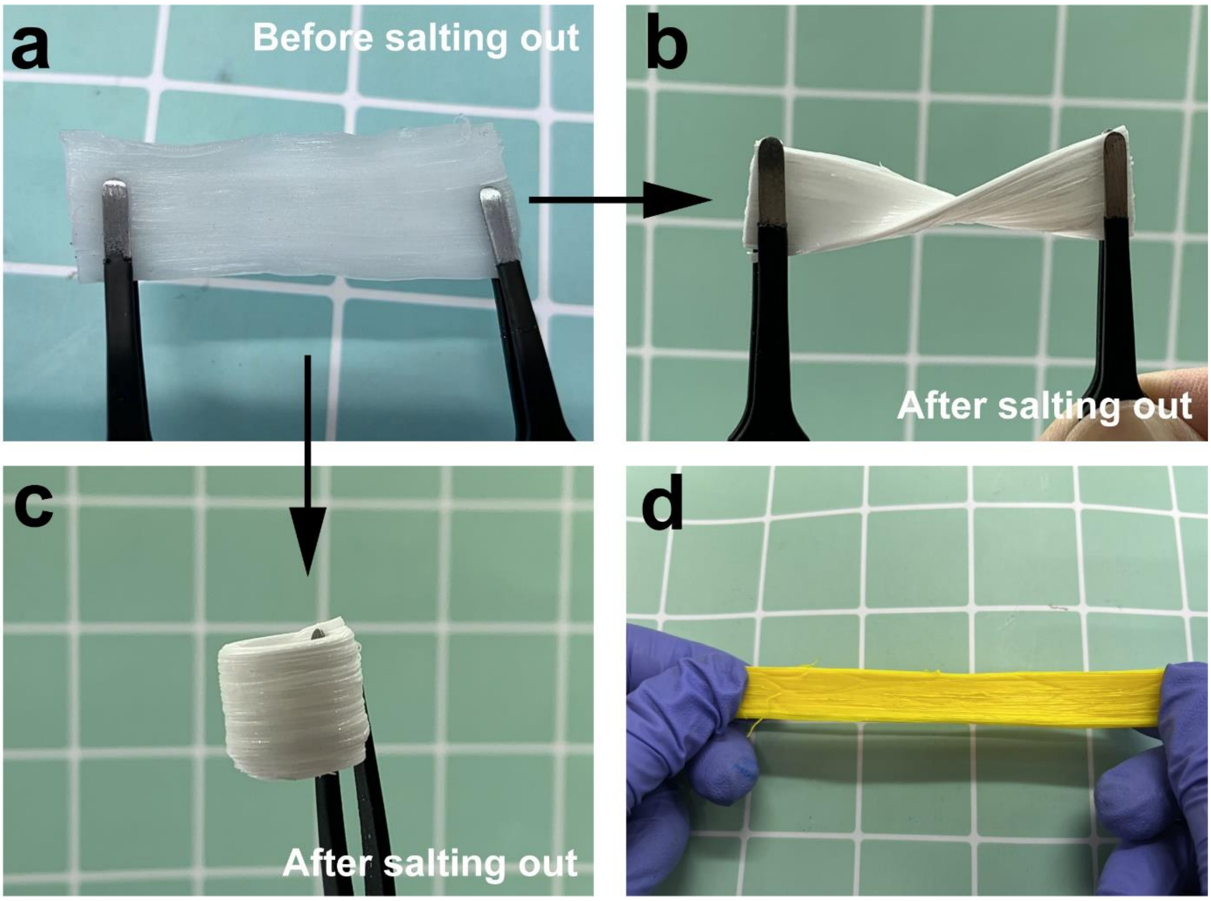

Supplementary Figure 7. Optical photographs of AFH (a) before salting out, (b-c) after salting out, (d) with 0.1 wt% fluorescein sodium salt.

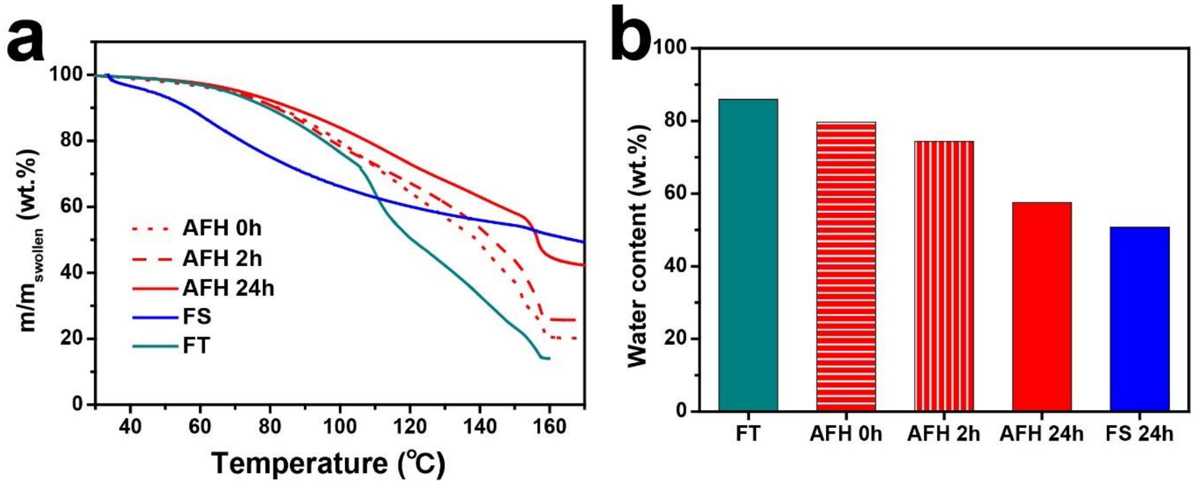

Supplementary Figure 8. The water content of the resulting hydrogels. (a) Thermal gravimetric analysis (TGA) curve of the FT hydrogel, FS hydrogel, and AFH (salting out for 0h, 2h, and 24h). $m/m_{swollen}$ represents the mass of the hydrogel samples during TGA measurement. (b) The water content of hydrogels was obtained from TGA results.

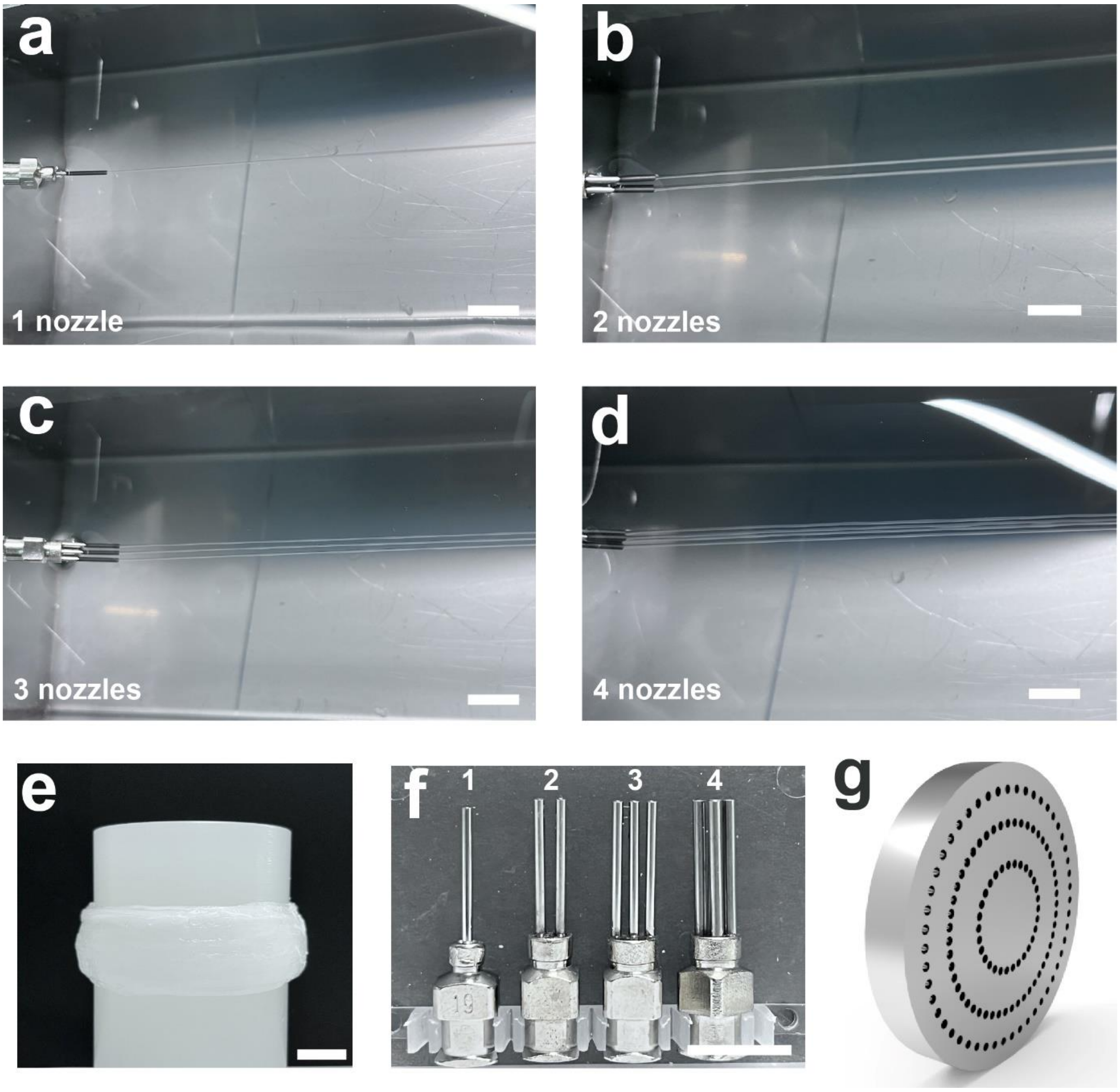

Supplementary Figure 9. Effect of different nozzles number on spinning efficiency. (a-d) The number of nozzles is gradually increased from one to four. Scale bar = 1 cm. (e) Anisotropic hydrogel obtained by spinning 20ml PVA precursors. Scale bar = 1 cm. (f) Spinnerets with different nozzles number. Scale bar = 1 cm. (g) Spinneret with a large number of nozzles for industrial wet spinning.

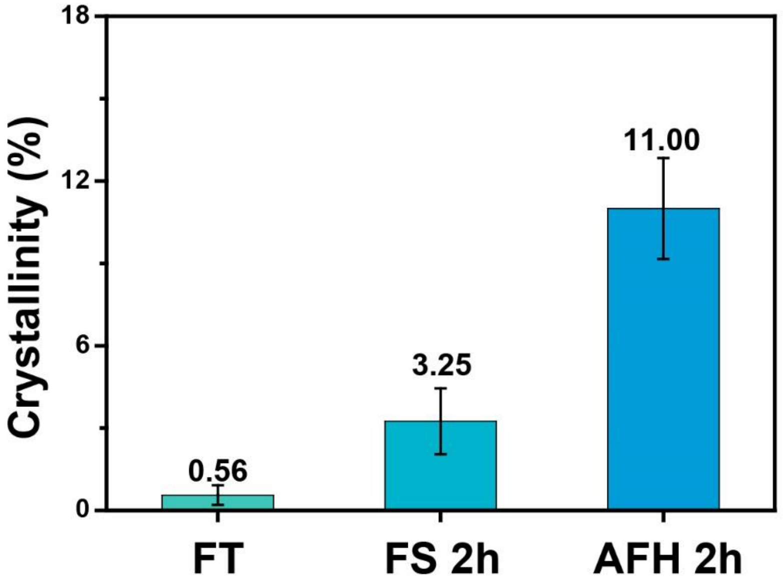

Supplementary Figure 10. Summarized crystallinities in the dry state of FS hydrogels and AFH after 2 h of salting out. Crystallinity data are presented as mean values ± SD, n = 3 independent samples.

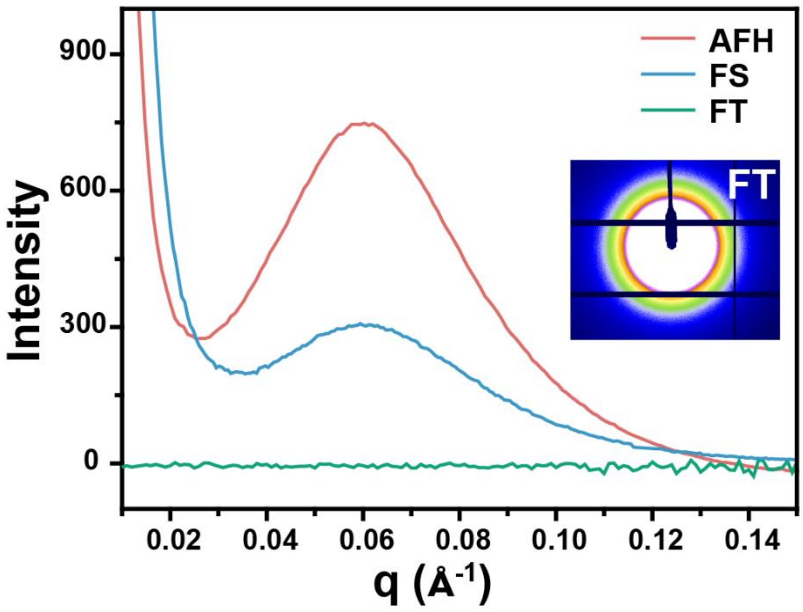

Supplementary Figure 11. The SAXS profiles of FT hydrogel, FS hydrogel, and AFH.

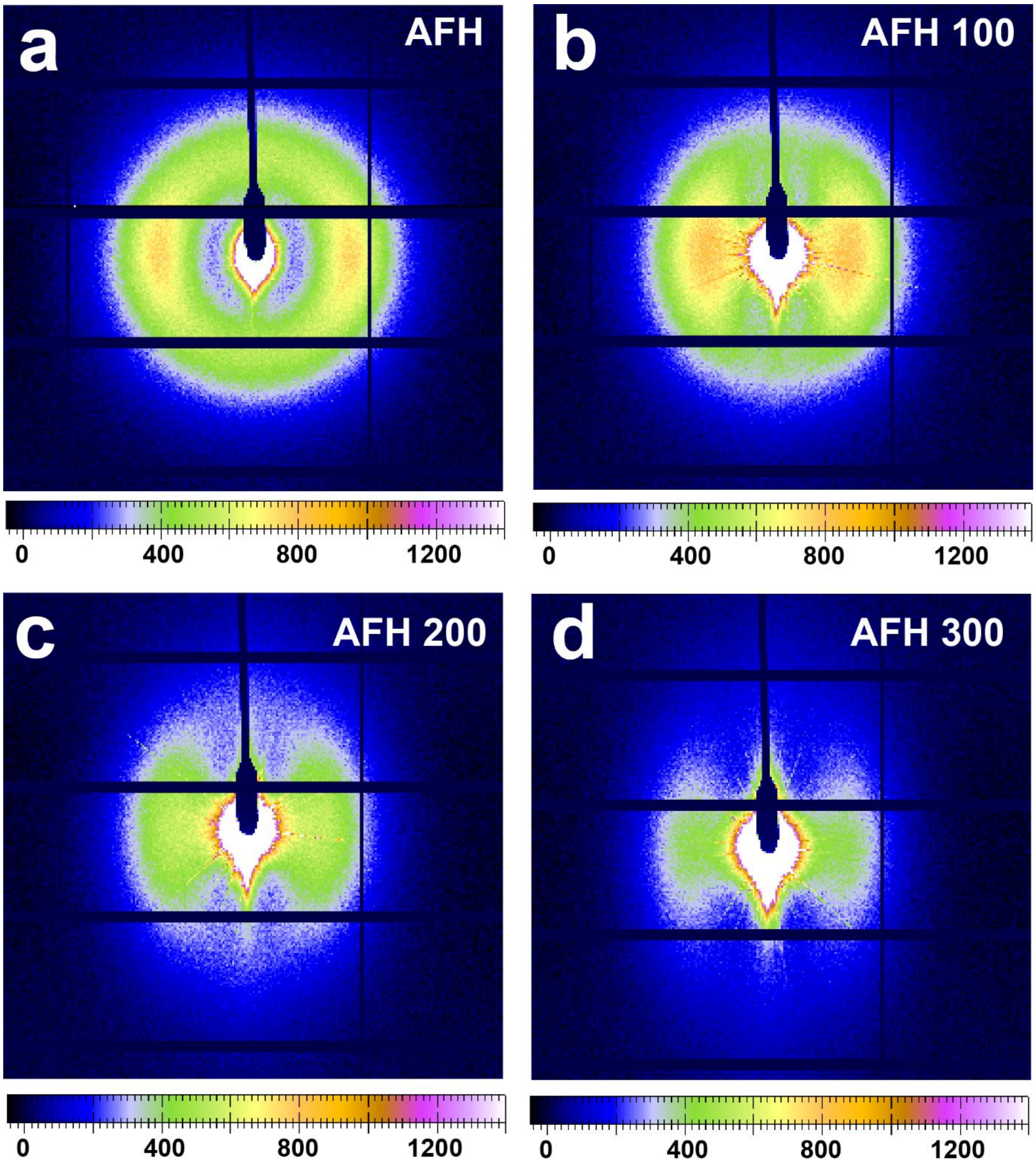

Supplementary Figure 12. 2D SAXS patterns when the tensile strains of AFH are (a) original state (AFH), (b) 100% (AFH 100), (c) 200% (AFH 200), and (d) 300% (AFH 300).

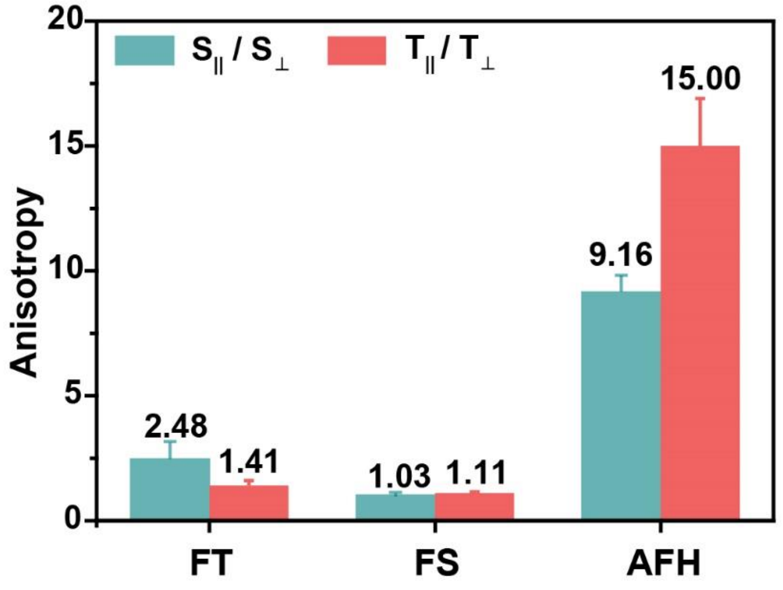

Supplementary Figure 13. The average result of strength anisotropy and toughness anisotropy of the FT hydrogels, FS hydrogels, and AFH. To evaluate the anisotropy of the resulting hydrogels, the tensile strength in the L direction was divided by the tensile strength in the R direction ($S_{\parallel}$ / $S_{\perp}$), and the toughness in the L direction was divided by the toughness in the R direction ($T_{\parallel}$ / $T_{\perp}$). Tensile data are presented as mean values ± SD, n = 3 independent samples.

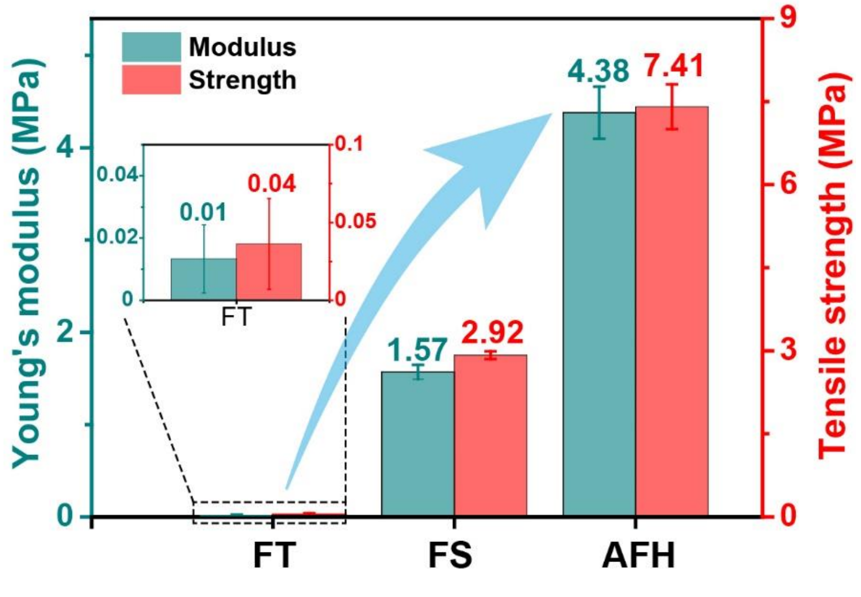

Supplementary Figure 14. The average result of Young's modulus and tensile strength of the FT hydrogels, FS hydrogels, and AFH after 24 h of salting out. Tensile data are presented as mean values ± SD, n = 3 independent samples.

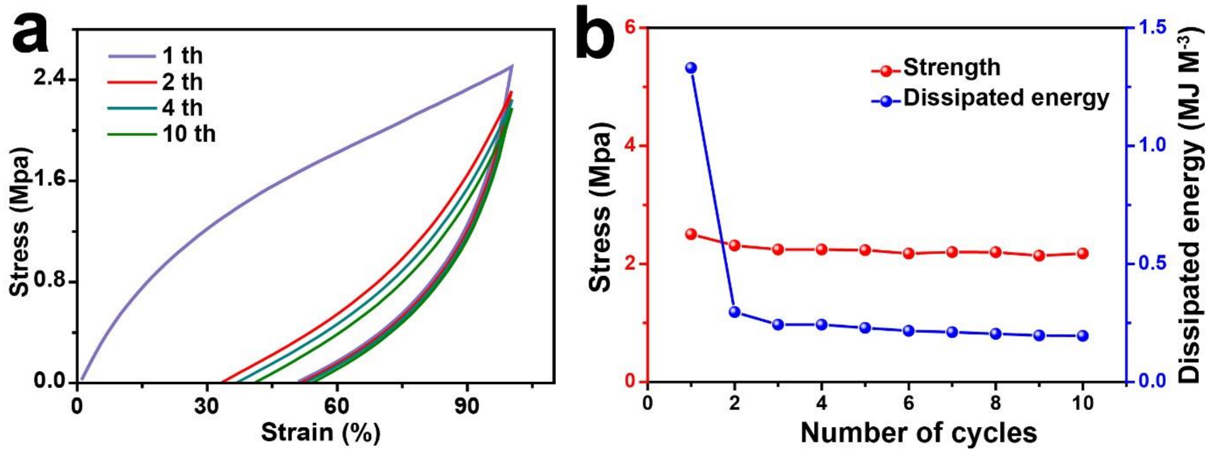

Supplementary Figure 15. (a) Cyclic stress-strain curves of the AFH under a constant strain (100%). (b) The corresponding tensile strength and dissipated energy of the AFH.

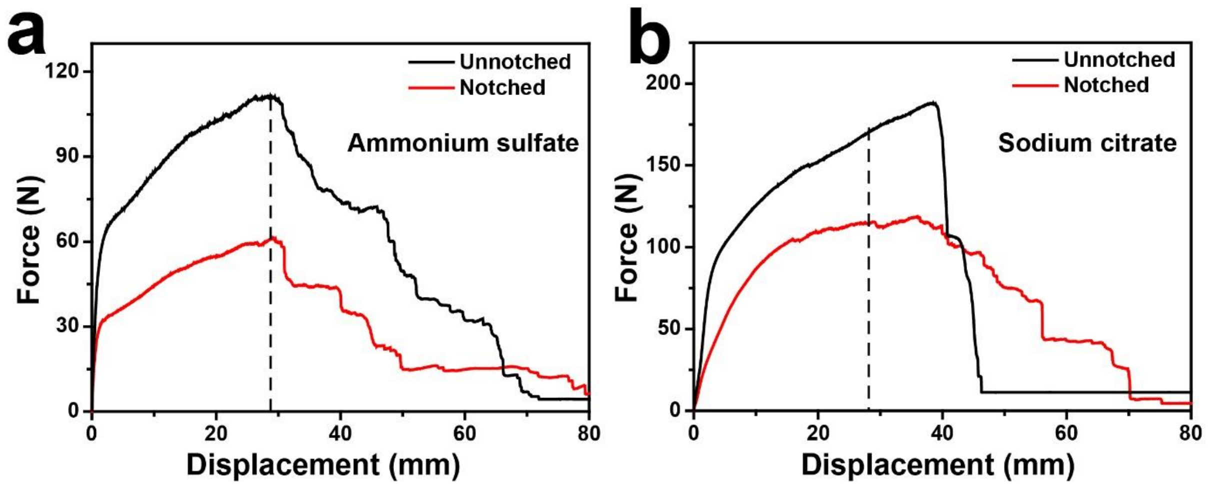

Supplementary Figure 16. The force-displacement curves of unnotched and notched AFH after 24 h of salting out in (a) ammonium sulfate and (b) sodium citrate

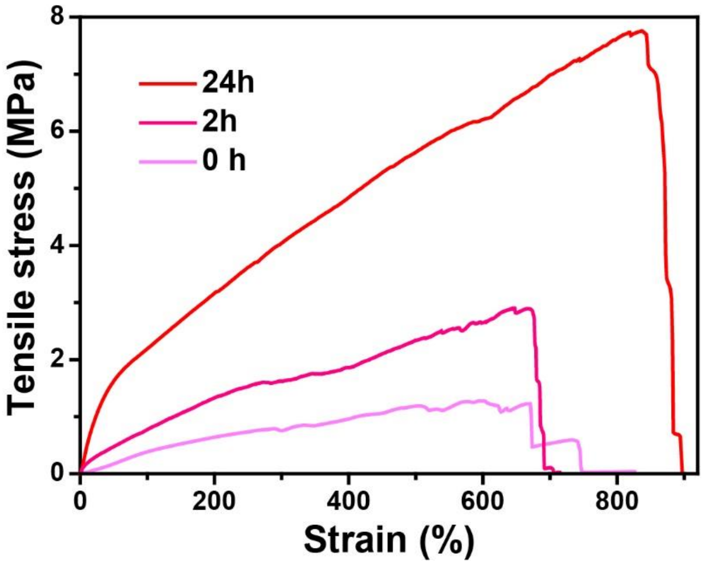

Supplementary Figure 17. The stress-strain curves of AFH after 0, 2, and 24 hours of salting out treated with ammonium sulfate.

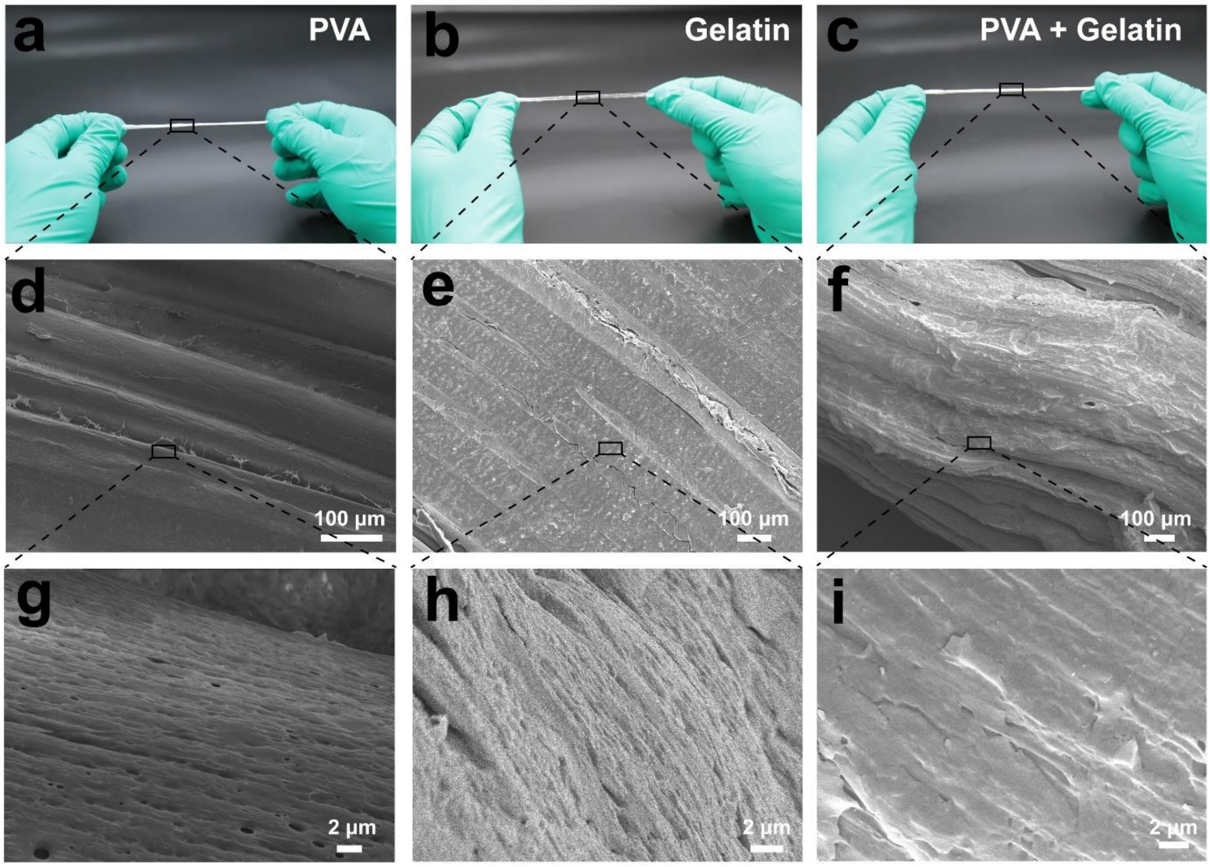

Supplementary Figure 18. SEM images of PVA, gelatin, and composite hydrogel of PVA and gelatin prepared by flow-induced alignment.

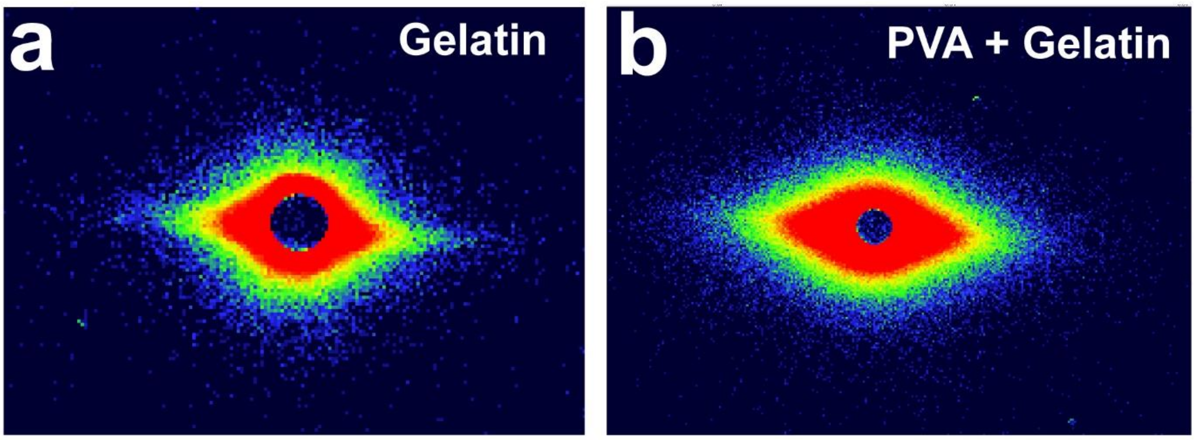

Supplementary Figure 19. 2D SAXS patterns of gelatin hydrogel and composite hydrogel of PVA and gelatin prepared by flow-induced alignment.

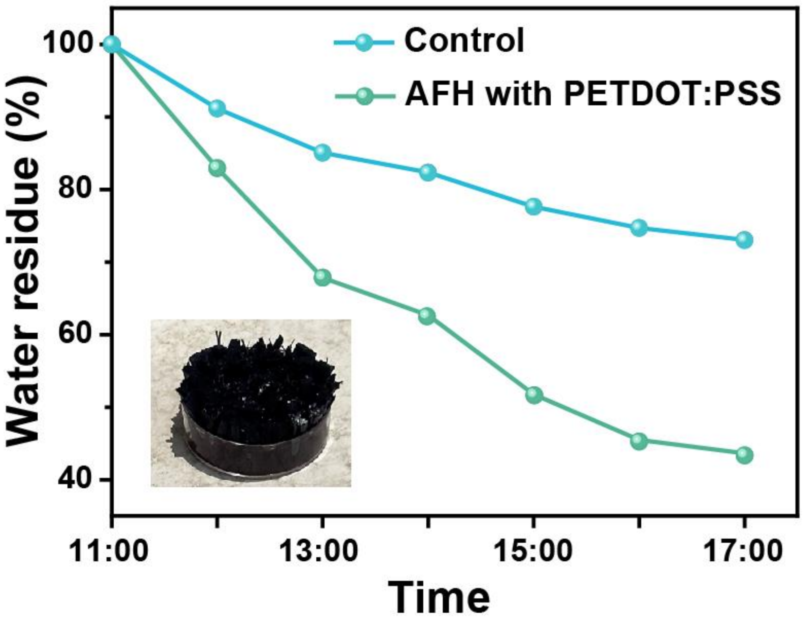

Supplementary Figure 20. Rapid water evaporation of AFH evaporator under natural light irradiation compared with a control group (without evaporator).

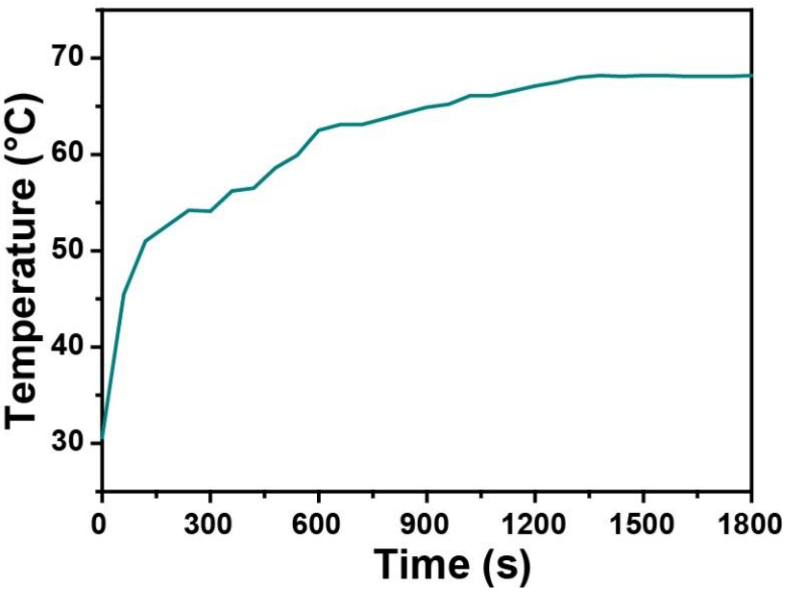

Supplementary Figure 21. The temperature of the sample over time under the illumination of an artificial light source.

Supplementary Table

Supplementary Table 1. The efficiency of the flow-induced alignment of nanofibrils.

Number of nozzles	Injection volume (ml)	Injection speed (ml min^{-1})	Nozzle diameter (mm)	Spinning time (min)
1	20	0.6	0.65	33.33
2	20	1.2	0.65	16.66
3	20	1.8	0.65	11.11
4	20	2.4	0.65	8.33

Supplementary Table 2. Elimination rates of BOD_5, COD, and heavy metal ion by using AFH hydrogel evaporators.

Sewage type	Category	Concentration before purification (mg L^{-1})	Concentration before purification (mg L^{-1})	Elimination rate (%)
Domestic sewage	BOD_5	12900	9.88	99.91
	COD	33900	41.00	99.88
Heavy metal sewage	Barium ion	17.4	0.004	99.98

Supplementary Methods

Modeling details and additional simulation results of PVA solutions in shear and elongation flow.

In the experiment, the PVA polymer with a molecular weight of 146,000 (kDa) had a degree of polymerization of about 3318. We coarse-grained (CG) the PVA polymer molecules to be of 100 monomers, where each CG monomer consisted of 33 repeat units in a real PVA chain. The size of the CG monomer was calculated by the OPLSAA all-atom force field for the PVA chain of 33 repeating units, which yielded a value of about $\sigma_0 =$ 3 nm. To keep the water and polymer beads at the same diameter $\sigma = 1\sigma_0$, 472 water molecules were modeled as one coarse-grained solvent bead. The open-source software LAMMPS was employed for all the MD simulations and calculations.

The bonded interactions between neighboring segments along chain contours of coarse-grained PVA molecules were represented by a finite extensible nonlinear elastic (FENE) potential[1-2]:

$$U_{fene} = -0.5\kappa R_0^2 \, ln\left(1 - \left(\frac{r}{R_0}\right)^2\right) + 4\varepsilon\left[\left(\frac{\sigma}{r}\right)^{12} - \left(\frac{\sigma}{r}\right)^6\right]$$

where R_0 = $1.5\sigma_0$ and κ = $30.0\varepsilon_0 \, / \, \sigma_0^2$ were selected for all simulations to prevent chain crossings. The nonbonded interactions between all segments were modeled as a full-range Lennard-Jones (LJ) potential.

$$U_{lj}(r) = 4\varepsilon\left[\left(\frac{\sigma}{r}\right)^{12} - \left(\frac{\sigma}{r}\right)^6 - \left(\frac{\sigma}{r_c}\right)^{12} + \left(\frac{\sigma}{r_c}\right)^6\right]$$

The energy strength between monomers, which was ε_{mm}, was set to 0.5 to simulate an aqueous solution environment. Here r_c was the cut-off distance of the interaction, fixed at r_c = $2.5\sigma_0$ for all simulations. In the following, the diameter of the PVA monomer bead was set to $1\sigma_0$. In experiments, the tensile rate of the PVA solution was 3.14 m min^{-1}, which could be transformed to 6.99 $\sigma_0 \, / \, \tau_0$ in our CG simulation, where τ_0 was the time unit in CG simulations. The simulation box containing 100 polymer chains was fabricated with a volume ratio of 60%, 45%, and 34%, respectively.

Elongation flow of PVA polymer solutions. As shown in Supplementary Figure 22(a-b), when a uniaxial tensile deformation was applied to the simulation box, the mean square end-to-end distance increased along the strain orientation. Supplementary Figure 23(a-c) demonstrated that the elongation flow-induced orientation of the PVA polymers stayed nearly the same by changing the volume fractions of polymers.

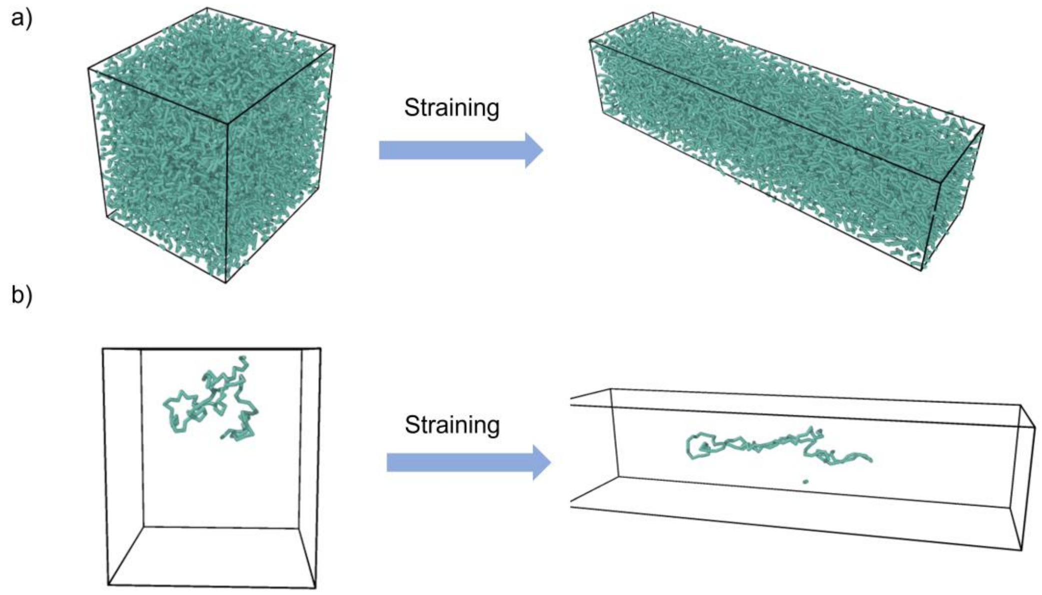

Supplementary Figure 22. (a) Snapshots of the stretch process at distinct times of PVA solution. (b) The snapshots of one PVA chain in PVA solution at the corresponding times in panel (a).

We also considered the effect of temperature on the deformation of PVA polymers. The volume ratio of the polymer was set to 0.61 and stretched at different temperatures, see Supplementary Figure 23(d-f). It was clearly shown that the elongation-induced deformation did not change as the temperature varied.

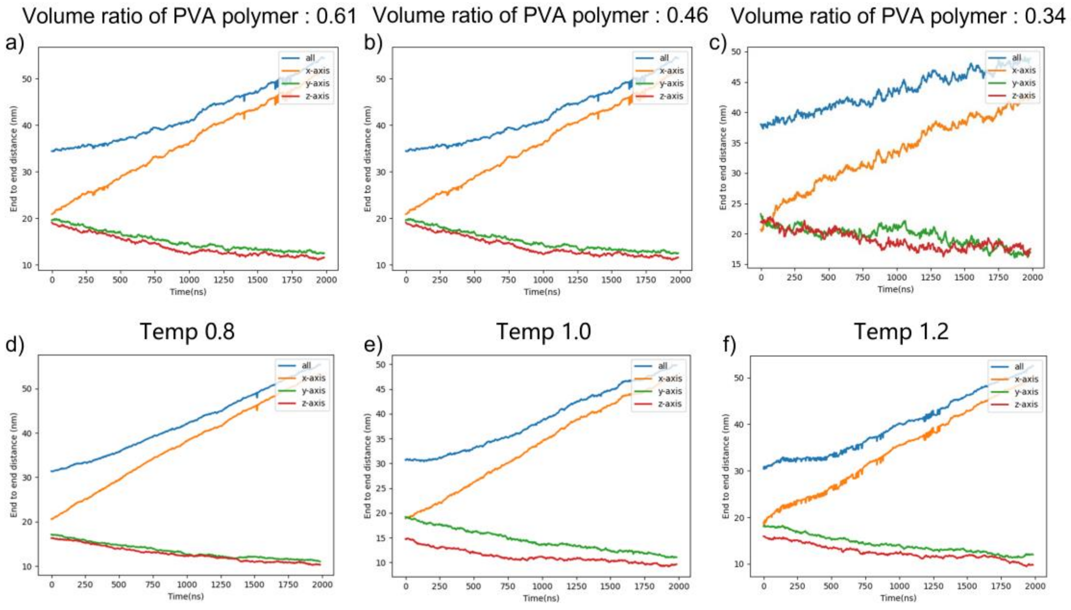

Supplementary Figure 23. Time-dependent end-to-end distance of PVA chains at different volume ratios of (a) 0.61, (b) 0.46, (c) 0.34 of the same temperature T = 1.4, and at distinct temperatures 0.8, 1.0, 1.2 of the same volume ratios of 0.61 during the x-axis stretching.

Shear flow of PVA polymer solutions. To model the shear effects, we include the solvents explicitly, and the energy strengths between water-water ε_{ww}, monomer-monomer ε_{mm}, and water-monomer ε_{wm} were set to be $\varepsilon_{ww} = \varepsilon_{mm}$=0.5 and $\varepsilon_{wm} = 0.6$ to keep the good solvent condition of PVA chains in solution. At low polymer concentration and shear rate, with a concentration of 10 wt% PVA aqueous solution and an injection speed of 0.6 ml min^{-1}, we included 15480 CG solvent beads and 100 polymers into one simulation box. The shear rate was fixed at 1.71 m min^{-1} in the experiment, which was 3.8 $\times 10^{-3}$ σ_0/τ_0 in the CG model. At high polymer concentration and shear rate, with a concentration of 25 wt% PVA aqueous solution and injection speeds of 1.75 ml min^{-1} and 3.5 ml min^{-1}, we included 4944 CG solvent beads and 100 polymers into one simulation box. The linear speed was fixed at 5 m min^{-1} and 10 m min^{-1}, which were 1.1$\times 10^{-2}$ σ_0/τ_0 and 2.2 $\times 10^{-2}$ σ_0/τ_0 in the CG model. Supplementary Figure 24 and Supplementary Figure 25 showed the snapshots and the changes of mean square end-to-end distances concerning time at temperatures 0.8, 1.0, 1.2, and 1.4 (At low polymer concentration and shear rate).

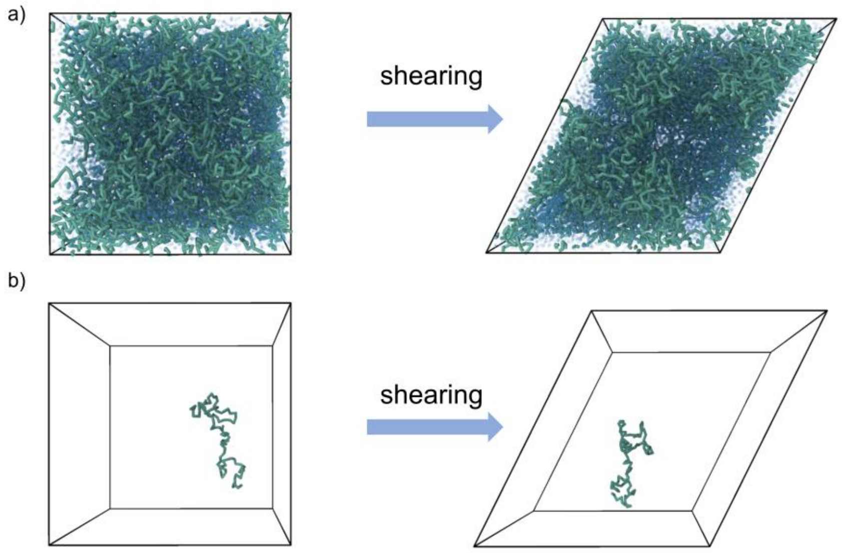

Supplementary Figure 24. (a) Snapshots of the shear process of PVA solutions at distinct times. (b) The snapshot of one PVA chain in PVA solution at the corresponding time in panel (a).

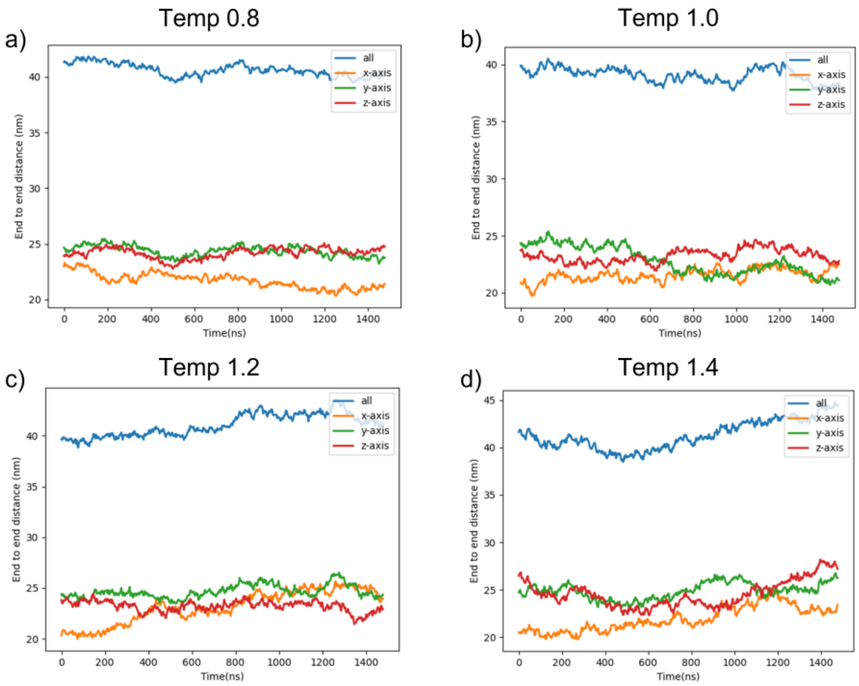

Supplementary Figure 25. The mean square end-to-end distance and its Cartesian coordinate components as a function of time during the same shear rate at distinct temperatures (a) 0.8, (b) 1.0, (c) 1.2, (d) 1.4.

Supplementary References

1 Grest, G. S. & Kremer, K. Molecular dynamics simulation for polymer in the presence of a heat bath. *Phys. Rev. A* **33**, 3628-3631 (1986)

2 Tang, Q., Rossner, C., Vana, P. & Müller, M. Prediction of Kinetically Stable Nanotheranostic Superstructures: Integral of First-Passage Times from Constrained Simulations. *Biomacromolecules* **21**, 5008-5020 (2020)
