# Supplementary material for: Bioinspired structural hydrogels with highly ordered hierarchical orientations by flow-induced alignment of nanofibrils
Source: Nat Commun. 2024 Jan 2;15:118. doi: 10.1038/s41467-023-44481-8 (PMC10761753; doi:10.1038/s41467-023-44481-8)
Supplement: Supplementary file 3 — Description of Additional Supplementary Files [file 41467_2023_44481_MOESM3_ESM.pdf]

Description of Additional Supplementary Files

File Name: Supplementary Movie 1
Description: Flow-induced orientation of PVA chains during the spinning process.

File Name: Supplementary Movie 2
Description: Damage-tolerant architecture of the precut AFH

File Name: Supplementary Movie 3
Description: Water transport of AFH.

File Name: Supplementary Movie 4
Description: Water transport of FT hydrogel and FS hydrogel.
